# Snacking pattern of college students in Saudi Arabia: a cross-sectional study

**DOI:** 10.1186/s40795-022-00544-5

**Published:** 2022-05-19

**Authors:** Israa M. Shatwan, Najlaa M. Aljefree, Noha M. Almoraie

**Affiliations:** Food and Nutrition Department, Faculty of Human Sciences and Design, King Abdulaziz University, Jeddah, 3270 Saudi Arabia

**Keywords:** College students, Snacking pattern, Sweet, Fast food, Healthy snack, Beverages, Dairy products

## Abstract

**Background:**

Although unhealthy snack foods are commonly consumed by college students, snacking patterns among college students have not been comprehensively examined in Saudi Arabia. In our study, we aimed to investigate snacking patterns among Saudi college students and to assess sociodemographic data that affect adherence to these snacking patterns.

**Methods:**

Between January 2021 and March 2021 in Jeddah, Saudi Arabia, an online survey was conducted with 662 college students from a Saudi university. The survey included sociodemographic questions and a short food frequency questionnaire (FFQ) regarding the consumption of common snack foods. Snacking patterns were generated from the FFQ using the factor analysis method.

**Results:**

Factor analyses generated seven snacking patterns, which explained 64.5% of the variance in snacking. Students in their early academic years (1–3 years) had a higher score for the convenience snack and fast-food pattern (0.22 ± 0.48 and 0.31 ± 0.52, respectively) than senior students (4–6 years) (*P* = 0.03 and 0.04, respectively). Healthy snacks patterns were higher among students at higher income levels (*P* = 0.006) and active students (*P* < 0.001) than among students at low- or mid-income levels and inactive students. Higher adherence to the beverages pattern was observed among male students (*P* = 0.03), active students (*P* = 0.01), and students with obesity than their counterparts (*P* = 0.02). The dairy products pattern was higher among male students (*P* = 0.04), students at higher income levels (*P* = 0.04), and students with obesity (*P* = 0.03) than their counterparts.

**Conclusions:**

Most snacking patterns identified among the study participants were considered unhealthy. Adherence to healthy snacks is influenced by physical activity and family income. These findings may be helpful in the future for developing adequate nutrition education programs that promote health by adhering to healthy snack choices in this critical age group. Further studies are needed to confirm these findings and to investigate snacking patterns among other age groups in Saudi Arabia.

## Background

Saudi Arabia, along with other Arabic Gulf countries, has witnessed massive economic development in the last decades, which has negatively influenced the eating patterns and lifestyle of the Gulf population [1]. The growth in socioeconomic status has led to enormous changes in dietary patterns to become more a westernized unhealthy diet, which consists of high amounts of sugar, saturated fat, and processed food. In addition, economic growth has considerably influenced lifestyle behaviors, resulting in a lack of physical activity and adoption of a sedentary lifestyle. The vast changes in eating patterns included a high intake of fast food, fried food, and energy-dense snacks. Subsequently, the shift in diet and lifestyle has resulted in a high increase in non-communicable diseases, such as obesity and type 2 diabetes, among these populations [1–3].

Snack foods were mostly considered unhealthy eating habits in previous research [4, 5]. Food items that are high in fat, sugar, and total calories, such as chocolate and potato chips, are commonly consumed as snacks [6]. Prior research has shown that snack intake may be associated with obesity and metabolic syndrome [7–9]. Other studies have reported contradictory findings [10]. Indeed, various definitions and strategies have been used to define and measure snacks in previous research, which has resulted in contradictory findings regarding the effect of snacks on health [11]. Snack intake is a common global behavior among young adults. For example, daily snack consumption was highly prevalent among university students in Lebanon (50%), China (30%), Malaysia (50%), India (54%), and Saudi Arabia (30%) [6, 10, 12–14]. College students are at an age when many changes take place in their lives, including spending a lot of time away from home and staying at university campuses. This transition mainly influences eating habits, undesirably contributing to weight gain, which may have significant consequences in the future, as weight gain in young adults’ life span is considered a considerable risk factor for obesity in late adulthood. Thus, promoting healthy eating habits is crucial to this population, as they would carry on these behaviors in the future [15–17].

Prior research was mostly focused on the impact of a particular nutrient or food group on illness; however, assessing the influence of overall dietary patterns generate accurate and comprehensive findings on the nutritional status and its relationship with various illnesses. Factor analysis is a method used to deduce dietary patterns by grouping variables that have identical attributes together; thus, conceivably explaining the overall nutritional status of a particular population [18]. Consequently, assessing snacking patterns among college students using factor analysis is crucial to exhibit the intricacy of the intake of various food groups and may possibly produce important nutritional recommendations. In Saudi Arabia, despite the high intake of snacks among young adults, snacking patterns among college students have not been comprehensively examined. Therefore, the current study aimed to investigate snacking patterns among Saudi college students and to examine their association with sociodemographic characteristics.

## Methods

### Ethics statements

The study protocol was approved by the Biomedical Ethics Research Committee of King Abdul-Aziz University (reference number: 9-21) and was conducted in accordance with the Declaration of Helsinki. All participants were informed of the study purpose and signed a consent form.

### Study design and participants

This cross-sectional survey was conducted between January 2021 and March 2021 in Jeddah, Saudi Arabia to investigate snacking patterns among Saudi college students. According to the Deanship of Admission and Registration at King Abdulaziz University, the total number of students in 2019 was 80,000 [19]. The sample size calculator suggested that the required sample size to achieve sufficient statistical power was 659, which was based on a response distribution of 50%, a 5% margin error, and 99% confidence level [20]. The inclusion criteria were college students, full-time students for all academic years, students aged between 18 and 29 years, and students of both sexes. Pregnant or breastfeeding women, students on any form of medication, and students with any known chronic diseases were excluded. Any participants who provided missing information on FFQ were excluded; however, all 662 college students fully completed the questionnaire. The survey was administered via an Internet questionnaire platform.

### Demographic and lifestyle questionnaires

The students were recruited via university e-mail, and the invitation sheet demonstrated the essential statement outline of the study and a link to the online survey. Data was collected through an online survey, whereas the teaching was conducted both on campus and online because of the COVID-19 pandemic. We collected each student’s general information, such as sex, age, academic year, monthly household income (< 5000, 5000–15,000, > 15,000 SR), and physical activity (inactive or active). The questionnaires have been described in detail elsewhere [21].

### Anthropometric data

All height (cm) and weight (kg) measurements were self-reported. Body mass index (BMI: weight/height^2^, kg/m^2^) was determined and classified according to the World Health Organization assessment [22].

### Food frequency questionnaire

Consumption of snack foods and beverages that included 27 food items was collected using a validated and reliable food frequency questionnaire (FFQ) [16]. The definition of snacks was written to ensure that students knew what we meant. Participants were asked about their consumption over the past month, according to a 9-grade scale (Never or less than once a month/ 1-3 times a month/ Once a week/ 2-4 times a week/ 5-6 times a week/ Once a day/ 2-3 times a day/ 4-5 times a day/ More than 6 times a day).

#### Food categories

Snack food items included in the FFQ were grouped according to the main food categories [11] as follows:

1. fruit and vegetables (3 items) including fruit juices, fruit, and vegetables.
2. caffeinated drinks (3 items) including energy drinks, coffee, and tea.
3. sweetened energy-dense beverages (2 items) including energy drinks and soft drinks.
4. sweets (5 items) including chocolate candy bars, cookies, pastries, pudding, and ice cream.
5. dairy products (6 items) including milk, alternative milk substitutes, chocolate milk drinks, Greek yogurt, regular yogurt, and cheese.
6. grains (4 items) including cereal, sandwiches, granola, and pasta.
7. fast food (4 items) including burgers, fries, sausage, and pizza
8. chips.

Food category scores were calculated separately. The participants’ response for each food item was recorded according to the 9-grade scale (never or less than once monthly, 1–3 times per month, once per week, 2-4 times per week, 5-6 times per week, 1 time per day, or 2 -3 times per day, 4-5 times per day, more than 6 per day). Each item was assigned a score from 0 to 9, zero indicated never or less than once monthly and 9 indicated more than 6 times per day. The total possible score for each item was 9. Subsequently, the sum of the scores for all the items was divided by the number of items included in the category.

### Identification of dietary pattern

In the beginning, the Kaiser-Meyer-Olkin Measure (KMO) of Sample Adequacy and the Bartlett Test of Sphericity were used to assess data adequacy. The KMO and Bartlett’s sphericity tests confirmed the sample adequacy for factor analysis (KMO = 0.84 and *P* < 0.001, respectively). To generate dietary patterns, 27 snack food items were entered into factor analysis (principal component) [3, 23]. The 27 items were fruit juices, fruit, vegetables, energy drinks, coffee, tea, soft drinks, chocolate candy bars, cookies, pastries, pudding, ice cream, milk, alternative milk substitutes, chocolate milk drinks, Greek yogurt, regular yogurt, cheese, cereal, sandwiches, granola, pasta, burgers, fries, sausage, pizza, and chips. For entry into the factor analysis, food groups may be entered by absolute weight in grams, the number of servings, or percentage of energy intake [24]. Given this, we decided to consider the food variables in terms of the number of servings per day. Thus, the frequency of intake was used to estimate the average of servings per day of each food item consumed, in order to use it in factor analysis.

Orthogonal varimax rotation was applied to obtain factors so that the factors (snacking patterns) were uncorrelated with each other and were easier to interpret. The orthogonal varimax rotation helps to detect specific structure in the relationships between variables; thus, food variables remain independent of each other. It also reduces the cross-loading of variables [25]. The eigenvalue and scree plot were applied to decide which factors remained. After evaluating the eigenvalues, snack food items with factor loadings ≥0.3 were used to interpret each snacking pattern while eigenvalues > 1 were retained. Labeling of the seven snacking patterns was primarily descriptive and based on our interpretation as well as the snack food items with the highest loadings [26]: convenience snack pattern, fast food pattern, sweet snack pattern, healthy snacks pattern, beverages pattern, dairy products and substitutes pattern, and caffeine drink pattern.

### Statistical analysis

Results are presented as the mean ± standard deviation of the scores. Significant differences in the demographics of the college students across food category scores were assessed using the independent t-test, except for family income, in which one-way analysis of variance was used. Normality distribution was checked for variables and log 10 was applied for skewed data. In addition, an analysis was conducted to estimate the average adherence to the seven snacking patterns identified by factor analysis using a univariate linear regression model. All analyses were performed using SPSS software, version 28 (IBM Corp.). Statistical significance was set at *P* < 0.05.

## Results

### Participants’ characterization based on the eight snacking categories

In this study, 662 college students from King Abdulaziz University were included. The eight snacking categories and sociodemographic data of the colleges are presented in Table 1. Men had a higher consumption (2.60 ± 1.58) of sweetened energy-dense beverages than women (2.03 ± 1.16; *P* < 0.001); however, women had a higher consumption (2.75 ± 1.00) of sweets than men (2.46 ± 1.05, *P* = 0.01). Significantly higher consumption of fruit and vegetables (*P* = 0.007) and fast food (*P* = 0.01) was observed among higher-income college students than among middle- and low-income students. Active students consumed more from fruit and vegetables (3.34 ± 1.44) and dairy products (2.28 ± 0.93) than inactive students (2.76 ± 1.22 and 1.96 ± 0.73, respectively, *P* < 0.001). Students with obesity had a higher consumption of caffeinated drinks (*P* = 0.006) and chips (*P* = 0.04) than those without obesity.

**Table 1:** Demographics of the college students by eight snacking categories

	Fruit and vegetables	caffeinated drinks	Sweetened energy dense beverages	Dairy products	Grains	Fast food	Chips	Sweet
Gender								
Male	3.03 ± 1.43	2.82 ± 1.46	2.60 ± 1.58	2.20 ± 0.94	2.01 ± 0.92	2.89 ± 1.45	1.71 ± 1.33	2.46 ± 1.05
Female	3.11 ± 1.38	3.05 ± 1.27	2.03 ± 1.16	2.14 ± 0.86	2.25 ± 0.93	2.88 ± 1.29	1.77 ± 1.34	2.75 ± 1.00
*P* value	0.59	0.09	< 0.001	0.63	0.06	0.93	0.62	0.01
Age								
18-21	3.14 ± 1.36	2.99 ± 1.28	2.05 ± 1.18	2.17 ± 0.87	2.22 ± 0.85	2.91 ± 1.30	1.73 ± 1.32	2.74 ± 1.01
22-25	3.05 ± 1.42	3.05 ± 1.33	2.20 ± 1.32	2.14 ± 0.86	2.21 ± 1.02	2.86 ± 1.33	1.81 ± 1.36	2.67 ± 1.02
*P* value	0.38	0.57	0.13	0.72	0.86	0.69	0.46	0.43
Academic years								
1-3	3.16 ± 1.41	3.05 ± 1.32	2.11 ± 1.24	2.19 ± 0.92	2.29 ± 0.99	2.92 ± 1.39	1.73 ± 1.33	2.71 ± 1.10
4-5	3.03 ± 1.36	2.98 ± 1.28	2.12 ± 1.26	2.12 ± 0.82	2.14 ± 0.87	2.85 ± 1.22	1.80 ± 1.34	2.71 ± 0.92
*P* value	0.22	0.46	0.96	0.38	0.08	0.46	0.54	0.96
Monthly household income (SR):*								
< 5000	2.93 ± 1.38	2.91 ± 1.20	2.03 ± 1.14	2.04 ± 0.79	2.25 ± 0.86	2.83 ± 1.44	1.78 ± 1.41	2.62 ± 1.05
5000-15,000	3.00 ± 1.31	3.01 ± 1.31	2.06 ± 1.19	2.18 ± 0.91	2.17 ± 0.85	2.75 ± 1.19	1.72 ± 1.24	2.66 ± 0.94
> 15,000	3.33 ± 1.47	3.09 ± 1.35	2.24 ± 1.37	2.19 ± 0.86	2.26 ± 1.07	3.09 ± 1.36	1.81 ± 1.41	2.82 ± 1.07
*P* value	0.007	0.40	0.16	0.41	0.59	0.01	0.78	0.09
Physical activity								
Inactive	2.76 ± 1.22	2.90 ± 1.29	2.22 ± 1.24	1.96 ± 0.73	2.12 ± 0.88	2.96 ± 1.22	1.69 ± 1.26	2.69 ± 1.03
Active	3.34 ± 1.44	3.11 ± 1.31	2.04 ± 1.25	2.28 ± 0.93	2.28 ± 0.96	2.83 ± 1.37	1.81 ± 1.39	2.72 ± 1.00
*P* value	< 0.001	0.05	0.06	< 0.001	0.07	0.22	0.23	0.67
Obesity status								
Non-obese	3.10 ± 1.41	2.92 ± 1.26	2.06 ± 1.16	2.13 ± 0.85	2.24 ± 0.94	2.91 ± 1.34	1.69 ± 1.31	2.70 ± 1.03
Obese	3.11 ± 1.35	3.22 ± 1.37	2.25 ± 1.41	2.21 ± 0.91	2.17 ± 0.91	2.83 ± 1.24	1.92 ± 1.38	2.73 ± 0.97
*P* value	0.99	0.006	0.07	0.41	0.52	0.45	0.04	0.67

Data presented as mean and standard deviation

*P* value was analyzed using independent t-test

* *P* value was analyzed using one way ANOVA

### Snacking patterns based on factor loading analysis

Factor analysis revealed seven major snacking patterns among the college students (Table 2), which explained 64.5% of the total variance in food intake. The first snacking pattern component was labelled as a convenience snack and had high positive factor loadings (≥0.3) for cheese, sandwiches, chips, pudding, cereal, ice cream, yogurt, pasta, pastry, and granola. The second was the fast-food pattern because it had a high factor loading towards the consumption of burgers, pasta, fries, and pizza, and a positive factor loading for cereal, ice cream, sweet milk, cookies, pastry, and soft drinks. The third snacking pattern had a high factor loading towards the consumption of candy, chocolate, cookies, pastry, sweet milk, soft drinks, and ice cream; thus, it was labelled the sweet snack pattern. The fourth snacking pattern was the healthy snack pattern because it had a high factor loading towards the consumption of vegetables, fruits, yogurt, and Greek yogurt. The fifth snacking pattern was beverages, which had a high positive factor loading for fruit juices, energy drinks, Greek yogurt, and sweet milk. The sixth was dairy products and substitutes because it had a positive factor loading towards the consumption of milk alternatives, milk, sweet milk, yogurt, and Greek yogurt. The last snacking pattern was caffeinated drinks, and it had a positive factor loading for coffee and tea. These snacking patterns among college students explained 14.5, 13.2, 11.6, 8.4, 6.2, 5.7, and 4.9% of the dietary intake variances, respectively.

**Table 2:** Factor loadings matrix of seven snacking patterns

	Convenience snack	Fast foods	Sweet snacks	Healthy snacks	Beverages	Dairy products and substitutes	Caffeinated drinks
Cheese	**0.827**	0.165		0.128			
Sandwiches	**0.787**		0.168	0.138			
Chips	**0.736**	0.184	0.219		0.158		
Pudding	**0.713**	0.234	0.102	0.168	0.131	0.110	
Cereal	**0.569**	**0.458**		0.156			
Ice cream	**0.518**	**0.353**	**0.504**				
Yogurt	**0.452**		0.226	**0.382**	−0.145	**0.410**	
Burger	0.126	**0.860**	0.318	0.104			
Pasta	**0.306**	**0.803**				0.117	
Fries	0.189	**0.742**	0.261	0.133	0.268		
Pizza	0.215	**0.699**	0.272		−0.123		0.103
Sweet Milk		**0.457**	**0.330**	−0.201	**0.334**	**0.428**	
Candy	0.231	0.218	**0.808**	0.117	0.127		
Chocolate	0.125		**0.795**				
Cookies	0.165	**0.332**	**0.765**	0.131			
Pastry	**0.405**	**0.329**	**0.525**	0.132	−0.131	0.284	0.159
Vegetables	0.203	0.104	0.100	**0.827**			
Fruit	0.152		0.157	**0.816**	0.110		
Fruit Juices			0.110	0.172	**0.693**		
Energy drinks				−0.151	**0.662**	0.218	0.188
Greek yogurt				**0.421**	**0.506**	**0.320**	
Milk alternative				0.111		**0.781**	0.126
Soft drinks	−0.209	**0.380**	**0.340**	−0.147	0.219	**0.528**	
Tea							**0.809**
Coffee	0.177	0.116	0.185			0.115	**0.737**
Milk	0.127			0.378	0.258	**0.698**	
Granola	**0.381**	0.143	0.142	0.399	0.175		
Total variance	14.5%	13.2%	11.6%	8.4%	6.2%	5.7%	4.9%

Factor loadings > 0.3 are highlighted in boldAbsolute values < 0.3 were excluded for simplicity

### Demographic data by snacking patterns

The coefficient Beta and 95% CI for coefficient Beta of the snacking pattern scores during last month based on the demographic data of college students from the general linear model are displayed in Table 3. Pattern 1 and 2, convenience snacks and fast food: students in early academic years (1–3 years) were associated with higher consumption of convenience snacks (*P* = 0.03) and fast food (*P* = 0.04) than senior students. Pattern 4, healthy snacks: students at higher income levels (*P* = 0.006) and active students (*P* < 0.001) had higher healthy pattern scores than students at low- or mid-income levels and inactive students. Pattern 5, beverages: higher pattern scores were associated with male sex (*P* = 0.03), active students (*P* = 0.01), and students with obesity (*P* = 0.02). Pattern 6, dairy products: male students (*P* = 0.04), students at higher income levels (*P* = 0.04), and students with obesity were associated with greater pattern scores. Pattern 7, caffeinated drinks, students with obesity had higher pattern scores than non-obese students (*P* = 0.05).

**Table 3:** Differences of the adherence of snacking pattern among 662 Saudi college students

	Convenience snack	Fast foods	Sweet snacks	Healthy snacks	Beverages	Dairy products and substitutes	Caffeinated drinks
Gender							
Male	Reference	Reference	Reference	Reference	Reference	Reference	Reference
Female	0.04 (−0.03 − 0.1)	0.001 (− 0.08-0.1)	0.01 (− 0.07-0.1)	0.01 (− 0.1-0.1)	-0.1 (− 0.2- -0.01)	− 0.09 (− 0.1- -0.002)	0.1 (− 0.02- 0.3)
*P* value	0.25	0.97	0.71	0.83	0.03	0.04	0.08
Age							
18-21	Reference	Reference	Reference	Reference	Reference	Reference	Reference
22-29	−0.02 (−0.07-0.04)	− 0.02 (− 0.08-0.04)	−0.02 (− 0.1 − 0.04)	-0.04 (− 0.1-0.03)	−0.01 (− 0.07-0.06)	−0.001 (− 0.06 − 0.06)	0.01 (− 0.1-0.1)
*P* value	0.52	0.53	0.53	0.25	0.87	0.98	0.81
Academic years							
1-3	Reference	Reference	Reference	Reference	Reference	Reference	Reference
4-5	−0.06 (−0.1- -0.003)	-0.06 (− 0.1- -0.003)	−0.05 (− 0.1- 0.01)	−0.06 (− 0.1- 0.01)	−0.01 (− 0.08- 0.05)	−0.03 (− 0.1- 0.03)	−0.04 (− 0.1-0.1)
*P* value	0.03	0.04	0.11	0.12	0.71	0.35	0.53
Monthly household income (SR):							
< 5000	Reference	Reference	Reference	Reference	Reference	Reference	Reference
5000-15,000	−0.04 (−0.1- 0.03)	−0.07 (− 0.1- 0.01)	−0.05 (− 0.1- 0.03)	0.03 (− 0.07- 0.1)	0.01 (− 0.08- 0.1)	0.0003 (− 0.09- 0.1)	0.07 (− 0.1- 0.2)
> 15,000	0.03 (− 0.05- 0.1)	0.006 (− 0.08- 0.1)	0.01 (− 0.08- 0.1)	0.1 (0.04- 0.1)	0.07 (− 0.02- 0.1)	0.1 (−0.003- 0.1)	0.1 (− 0.02- 0.3)
*P* value	0.09	0.05	0.18	0.006	0.22	0.04	0.22
Physical activity							
Inactive	Reference	Reference	Reference	Reference	Reference	Reference	Reference
Active	0.05 (−0.003- 0.1)	0.01 (−0.04- 0.08)	−0.01 (− 0.08- 0.05)	0.1 (0.1-0.2)	0.08 (0.01- 0.1)	0.06 (− 0.003- 0.1)	0.1 (− 0.03- 0.2)
*P* value	0.06	0.62	0.63	< 0.001	0.01	0.06	0.14
Obesity status							
Non-obese	Reference	Reference	Reference	Reference	Reference	Reference	Reference
Obese	0.02 (−0.03- 0.08)	−0.003 (−0.07-0.06)	−0.003 (− 0.07- 0.07)	0.03 (− 0.05- 0.1)	0.08 (0.01- 0.1)	0.07 (0.005- 0.1)	0.1 (− 0.005- 0.2)
*P* value	0.44	0.92	0.94	0.47	0.02	0.03	0.05

Data presented as Beta coefficient and 95% confidential interval

*P* value was analyzed using a univariate linear regression model

## Discussion

This study aimed to identify snacking patterns among university students in Saudi Arabia and to determine the sociodemographic characteristics of students favoring these snacking patterns. Our results indicated that male students consumed more sweetened beverages, while female students consumed more sweet snacks; students with higher income consumed more fruit and vegetables and fast food than those with middle- to low-income; and physically active students had higher intake of fruits, vegetables, and dairy products than inactive students. Seven snacking patterns were identified among the college students in this study. Moreover, the analysis showed heterogeneity in food choice among college students following these snacking patterns: students in early college years consumed more from the convenience and fast-food pattern, and higher adherence to the healthy snacking pattern was observed among physically active students and students at higher income levels. The beverages pattern was highly associated with male students, physically active students, and students with obesity. The dairy snacking pattern was highly associated with male students, students with high income levels, and students with obesity.

In our study, sex was associated with a higher consumption of unhealthy snack item, sweet beverages among male students and sweet snacks among female students. This finding is consistent with that of other studies in Bangladesh and Jordan, which showed that a higher consumption of sugar-sweetened beverages was associated with male students [27, 28]. Moreover, a cross-sectional study conducted among 387 undergraduate students, aged 18–25 years at a South African university, indicated that female students consumed more desserts, cakes, biscuits, chocolates, and sweets than male students [29]. In addition, findings from studies conducted among college students from Kuwait and European countries confirmed that women consumed more sweet snacks than men [30, 31]. A higher consumption of sugary food was found to be associated with an increased risk of obesity among college students [27].

The consumption of fast food, another unhealthy snack item, was linked to family income in our study. In line with this, higher socioeconomic class is one factor positively associated with frequent consumption of fast food according to a systematic review based on data of South Asian college students [32]. In contrast, fast-food consumption was not associated with higher income levels among college students from Pune [33] and Kuwait [34]. Increased consumption of fast food among college students could be related to limited time, convenience, and taste.

Regarding the association between obesity and the consumption of chips, a meta-analysis based on 18 cross-sectional studies demonstrated that higher sodium intake was associated with increased BMI [35]. In line with this, a large cohort of 6545 adolescents aged 12 to 19 years, from the United States showed that adolescents who were overweight or obese consumed more sodium from snacks compared to normal-weight adolescents [36]. However, a cross-sectional study showed that the increased consumption of salty snacks (including potato chips) was not associated with obesity among Serbian high school and college students [37].

Our study observed a higher consumption of caffeinated drinks among students with obesity than among those without. In line with this finding, overweight female college students from Riyadh had a significantly higher prevalence of coffee consumption than non-consumers [38]. Another study conducted on Chinese medical college students found that caffeinated drink consumption was significantly associated with increased body mass [39]. Among United States college students, the most consumed caffeinated drink was coffee, followed by energy drinks [40].

The consumption of healthy snack items, fruit and vegetables, was associated family income and physical activity in our study. Results from a cross-sectional study conducted among Kuwaiti college students showed that higher income was associated with increased consumption of vegetables among male students; however, the opposite was observed among female students [41]. Previous studies have confirmed that income has a positive impact on fruit and vegetable consumption [42, 43]. Regarding the level of physical activity, Dutch university students who did not adhere to physical activity guidelines had a low fruit and vegetable intake [44]. Data from 717 Brazilian college students showed that an extra 35 and 47 minutes per day of physical activity for men and women, respectively, was associated with an increase in one serving of fruits and vegetables [45]. Students that tend to adhere to a healthy lifestyle are more physically active and consume more fruits and vegetables.

Another healthy snack food that was associated with the level of physical activity among students was intake of the dairy products. A cross-sectional study conducted on 2610 individuals aged 18 to 59 years found that yogurt consumers were more physically active during leisure time [46]. Similarly, a study conducted on children and adolescents showed that higher levels of physical activity were associated with higher consumption of milk and dairy products in both boys and girls [47]. Dairy products are considered as a good source of nutrients that enhance the health and bone mass, which is important for athletes; thus students with higher physical activity tend to increase their consumption of dairy products.

Few studies have assessed snacking patterns using cluster or factor analysis. Among the Colombian population, packaged food (including sweets or candy, soft drinks, sausage, and fast food), fruit and vegetables/dairy, and traditional starch were identified as snacking patterns [3]. Another study conducted on adults in the United States found 10 patterns of snacking. These included cakes, cookies, pastries, sweet vegetables or legumes, alcohol, milk dessert, crackers or salty snacks, soft drinks, other grains, whole fruits, and coffee or tea [11]. Among Chinese children and adolescents, the major snacking patterns identified were fruits, dairy products, fruit products, and beverages [23].

In our study academic years were associated with two of unhealthy snack patterns, convenience, and fast-food patterns. A convenience dietary pattern (containing crisps and savory snacks, white bread, pasta, rice, and chips) was identified among 1448 university students in the United Kingdom. However, that dietary pattern was not in line with our study’s findings, as no association was confirmed between the convenience dietary pattern and academic years, and the mean score for students at year 4 and higher was higher than the mean score of students in their early years at a university [17]. Several previous studies have demonstrated a higher consumption of fast food among college students [34, 48, 49], but no correlation with academic year has been found. It could be that an increase academic stress in the later years of college lead to an increased consumption of convenience food among senior students.

Our results showed that male students, active students, and students with obesity had a higher consumption of beverages than their counterparts. A cross-sectional study conducted on 1138 college students showed that 68% of men consumed sugary drinks daily compared to 55% of women, and the sugary drinks included fruit juices, milk, soft drinks, and energy drinks [50]. Another study conducted among Polish adults showed that the consumption of sugar-sweetened beverages was higher among men than among women [51]. Data obtained from the Chinese National Survey among children and adolescents aged 6–17 years observed that children and adolescents with high physical activity had a higher consumption of sugar-sweetened beverages than those without, and a higher consumption of sugar-sweetened beverages was associated with abdominal obesity but not general obesity [52]. In contrast, children in the United States consuming high amounts of sugar-sweetened fruit drinks or soft drinks were at a higher risk of being overweight or obese [53].

Healthy snack pattern was associated with physical activity and family income. In line with our findings, a study conducted among university students from the United Kingdom aimed to assess their dietary pattern and confirmed a positive association between the health-conscious pattern (including fish, fruits, vegetables, oats, and low-fat yogurt) and physical activity level. Higher adherence to the health-conscious pattern was observed among very active students than among not very active students [17]. Previously, we mentioned that a higher income level increased the consumption of fruits and vegetables, which are the main factors in the healthy snack pattern.

Regarding the findings for the dairy product pattern, a previous study showed that male students had a higher mean consumption of dairy products than female students [54]. In addition, a higher income level was associated with a higher dairy product score [41]. The BMI of individuals is related to dairy product consumption [55]. However, a meta-analysis of randomized control trials and a longitudinal study demonstrated a beneficial effect of dairy intake on body weight [56, 57].

The present study has several strengths. First, to the best of our knowledge, this is the first study to report snacking patterns using factor analysis methods among college students in Saudi Arabia. The intake of snack food items was measured using the FFQ, which is considered appropriate for assessing dietary patterns, captures a longer period than the 24-hour recall, and is suitable for large sample sizes [17]. Additionally, a short food checklist is preferable to a long checklist because it reduces respondent fatigue and reporting errors. However, this study has some potential limitations that should be mentioned. Given the cross-sectional design of the study, the results could not establish causal associations. Additionally, the study sample was not representative of the different age groups in the population in relation to patterns of snack consumption, which may limit the generalizability of our findings. Future studies are needed to examine children and adolescents, and adults. Another limitation is that factor analysis involves factor analysis involves subjective decisions as determining factors included in dietary pattern based on eigenvalues, scree plots [58].

## Conclusions

This study provides insight into the snacking dietary patterns of Saudi college students along with their association with sociodemographic information. Seven snacking patterns were identified, some of which were considered healthy and unhealthy dietary habits. Family income and physical activity were shown to affect adherence to the healthy snacking pattern. These findings may be helpful in the future for developing adequate nutrition education programs that promote health by adhering to healthy snack choices in this critical age group. Moreover, further studies are needed to confirm these findings and to investigate snacking patterns among other age groups in Saudi Arabia.

## Data Availability

The datasets generated and/or analyzed during this study are not publicly available because of the use of data for further publications but are available from the corresponding author upon reasonable request.

## Abbreviations

BMI: Body mass index
FFQ: Food frequency questionnaire
